# Antioxidants Threaten Multikinase Inhibitor Efficacy against Liver Cancer by Blocking Mitochondrial Reactive Oxygen Species

**DOI:** 10.3390/antiox10091336

**Published:** 2021-08-24

**Authors:** Blanca Cucarull, Anna Tutusaus, Tania Hernáez-Alsina, Pablo García de Frutos, María Reig, Anna Colell, Montserrat Marí, Albert Morales

**Affiliations:** 1 Department of Cell Death and Proliferation, IIBB-CSIC, IDIBAPS, 08036 Barcelona, Spain; blanca.cucarull@gmail.com (B.C.); anna.tutusaus.lopez@gmail.com (A.T.); pablo.garcia@iibb.csic.es (P.G.d.F.); anna.colell@iibb.csic.es (A.C.); 2 Departament de Biomedicina, Facultat de Medicina, Universitat de Barcelona, 08036 Barcelona, Spain; 3 Digestive Unit, Hospital San Pedro, Rioja Salud, 26006 Logroño, Spain; taniahernaez@gmail.com; 4 Unidad Asociada (IMIM) IIBB-CSIC, CIBERCV, 08036 Barcelona, Spain; 5 Barcelona Clinic Liver Cancer (BCLC) Group, Liver Unit, Hospital Clínic of Barcelona, University of Barcelona, CIBEREHD, IDIBAPS, 08036 Barcelona, Spain; mreig1@clinic.cat; 6 CIBERNED, 08036 Barcelona, Spain

**Keywords:** chemotherapy, oxidative stress, glutathione, superoxide, BCL-2, hepatocellular carcinoma, tumor spheroids, mitochondria, apoptosis, mitophagy

## Abstract

Sorafenib and regorafenib, multikinase inhibitors (MKIs) used as standard chemotherapeutic agents for hepatocellular carcinoma (HCC), generate reactive oxygen species (ROS) during cancer treatment. Antioxidant supplements are becoming popular additions to our diet, particularly glutathione derivatives and mitochondrial-directed compounds. To address their possible interference during HCC chemotherapy, we analyzed the effect of common antioxidants using hepatoma cell lines and tumor spheroids. In liver cancer cell lines, sorafenib and regorafenib induced mitochondrial ROS production and potent cell death after glutathione depletion. In contrast, cabozantinib only exhibited oxidative cell death in specific HCC cell lines. After sorafenib and regorafenib administration, antioxidants such as glutathione methyl ester and the superoxide scavenger MnTBAP decreased cell death and ROS production, precluding the MKI activity against hepatoma cells. Interestingly, sorafenib-induced mitochondrial damage caused PINK/Parkin-dependent mitophagy stimulation, altered by increased ROS production. Finally, in sorafenib-treated tumor spheroids, while ROS induction reduced tumor growth, antioxidant treatments favored tumor development. In conclusion, the anti-tumor activity of specific MKIs, such as regorafenib and sorafenib, is altered by the cellular redox status, suggesting that uncontrolled antioxidant intake during HCC treatment should be avoided or only endorsed to diminish chemotherapy-induced side effects, always under medical scrutiny.

## 1. Introduction

Hepatocellular carcinoma (HCC) is often diagnosed at advanced stages with poor prognosis being the third leading cause of cancer death [1,2]. Despite recent advances in treatment, survival after HCC detection clearly needs to be improved and frequently depends on the efficacy of multikinase inhibitors (MKIs) [2,3]. In the last decade, most of the liver cancer patients have received sorafenib [4] as standard systemic therapy in first line, while regorafenib [5] and cabozantinib [6] has been prescribed for second line. Current treatments for HCC have been recently reviewed by Bruix et al. [7]. 

HCC has a complex genetic background, lacking specific driver mutations required for cancer cell survival. Therefore, metabolic weaknesses created in cancer cells by MKIs could be considered as an interesting opportunity for treatment in order to improve patient’s life expectancy [8]. Among them, mitochondrial alterations induced by MKIs, such as sorafenib, have received particular interest in cell death signaling [9,10,11]. In this sense, sorafenib, regorafenib and other MKIs have been shown to act by generating reactive oxygen species (ROS) from the mitochondrial respiratory system, inducing loss of mitochondrial membrane potential and changes in BCL-2 family proteins, which prime cancer cells to combinatory therapies with BH3-mimetics [12,13,14,15]. 

In clinical practice, MKIs are principally considered to act through specific tyrosine and threonine kinases and as anti-angiogenic compounds [2,3,16], while their mitochondrial effect and subsequent ROS production has been largely neglected as an important contributing mechanism until recently. For this reason, we wanted to examine if MKI-induced ROS play an important role in their anti-tumor activity and evaluate if changes in cellular antioxidants with relevant mitochondrial action may alter the response to cancer therapy in HCC treatment. 

Nutrition supplements are becoming familiar components of people’s diets around the world, and are most frequently used without the advice of a physician or healthcare provider [17,18]. In western countries, over-the-counter products are a common addition, particularly for individuals in special needs, such as sport practitioners, pregnant women, the elderly or those with chronic diseases [19,20]. Cancer patients are aware of their physical profile and frequently take dietary complements, principally vitamins and antioxidants. Among them, glutathione (GSH) and related precursors such as N-acetylcysteine or S-adenosylmethionine [21], antioxidant enzymes such as superoxide dismutase and mitochondrial protectors such as Coenzyme Q10 are frequently acquired for customers and individuals under cancer therapy. 

Therefore, we tested the effect of modulating GSH [21], as the main antioxidant involved in mitochondrial survival, on MKI efficacy and in superoxide levels, as the principal source of free radicals in the mitochondria [22], particularly after MKI exposure [10,23]. To do so, in in vitro cellular models and in 3D tumor spheroids, we studied relevant MKIs in the presence of the superoxide dismutase (SOD) mimetic MnTBAP [24] and GSH modulators, such as the inhibitor of GSH synthesis (BSO) or the permeable GSH supplier (glutathione methyl ester, GSHe). In addition, sorafenib is a well-known inducer of autophagy/mitophagy [25,26], and the participation of mitochondrial ROS in mitophagy induction is an emerging topic in different pathological conditions [27,28]. Therefore, we analyzed the potential influence of MKI-derived ROS in autophagy/mitophagy induction and their modulation depending on the cellular redox status.

Our work reveals that MKIs exhibit differential toxicity in hepatoma cell lines, an effect that is frequently potentiated after GSH depletion, promoting mitochondrial damage and mitophagy induction. Consistent with the important role of ROS in MKI anti-cancer activity, loading with specific antioxidants, such as the SOD mimetic MnTBAP or with GSHe, reduced chemotherapy efficacy in hepatoma cell lines and enhanced tumor growth in HCC spheroids. Our results suggest that mitochondrial ROS are critical in the anti-cancer activity of MKIs frequently prescribed for HCC treatment, while antioxidant compounds may alter MKI efficacy in HCC therapy and their uncontrolled consumption should be avoided during chemotherapy treatment.

## 2. Materials and Methods

### 2.1. Reagents

Dulbecco’s Modified Eagle’s Medium (DMEM), trypsin-EDTA, penicillin-streptomycin and dimethyl sulfoxide (DMSO), MTT (3-(4,5-dimethylthiazol-2-yl)-2,5-diphenyl tetrazolium bromide) (M2128), Hoechst 33258 (B1155) and DCF (D6883) were purchased from Sigma–Aldrich (St. Louis, MO, USA). All tissue culture-ware was from Nunc (Roskilde, Denmark). Proteinase inhibitors were from Roche (Madrid, Spain). ECL western blotting substrate was from Pierce (Thermo Fisher Scientific, Rockford, IL, USA). Novex Sharp Pre-Stained Protein Standard (LC5800) (T-3168) were from Invitrogen Life Technologies (Carlsbad, CA, USA). Sorafenib (BAY 43-9006, Nexavar) and Regorafenib (BAY 73-4506, Stivarga) are manufactured by Bayer. Cabozantinib and A-1331852 were purchased from MedChem Express (Monmouth Junction, NJ, USA). Buthionine sulfoximine, MnTBAP chloride and glutathione monoethyl ester were obtained from Santa Cruz Biotechnology (Dallas, TX, USA).

### 2.2. Cell Culture and 3D Tumor Liver Spheroid Generation 

Human liver tumor cell lines Hep3B, PLC/PRF/5 and HepG2 (European Collection of Animal Cell Cultures (ECACC)) were grown in DMEM (10% FBS) at 37 °C and 5% CO_2_. Hep3B cell spheroids were generated and plated in 96-well plates with a bottom coat of agarose [15], allowing spheroids to aggregate for 24 h before treatments. Tumor liver spheroids were kept at 37 °C and 5% CO_2_ for 7 days and growth was monitored daily.

### 2.3. Cell Viability

Cell viability was determined by the MTT assay; 1 × 10^4^ cells/well were seeded in a 96-well plate and incubated at 37 °C and 5% CO_2_. After treatments, 10 µL of MTT reagent (5 mg/mL) were added and incubated for 2 h. After removal of the medium, formazan crystals from dried plates were dissolved with 100 µL of 1-propanol. Absorbance was measured in a plate reader (Multiskan^®^ Spectrum, Thermo Fisher Scientific, Rockford, IL, USA) at 570 nm and 630 nm and cell viability calculated with untreated cells. 

### 2.4. Reactive Oxygen Species (ROS) Measurement 

Cellular ROS generation was quantified using dihydroethidium (DHE) probe that mainly targets the superoxide anion; 7.5 × 10^3^ or 1 × 10^4^ cells/well were seeded in 96-well plates. After treating cells with indicated drugs, DHE probe was added for 30 min. After probe internalization, 2 washes were performed with DMEM without phenol red and photos of 10 random fields taken using a Leica-CTR4000 microscope and LAS software.

### 2.5. Apoptotic Cell Death Detection 

Cells were seeded at 5 × 10^4^ cells/well in 12-well plates, treated for 8 h. Hoechst 33258 was added to the cell medium (10 µg/mL) for 30 min. After being washed, images of twelve random fields were taken using an Olympus IX-70 microscope with the CC-12 FW camera. After Hoechst staining, condensed nuclei were counted with ImageJ software. 

### 2.6. Immunofluorescence

Hep3B cells were seeded at a density of 50,000 cells/well in 12-well plates on 10 mm round coverslips. After treatments, cells were rinsed with PBS, fixed with 4% PFA for 15 min, washed with PBS kept, blocked in a solution of 1% fatty acid free BSA, 0.1% saponin and 0.5% glycine in PBS for 20 min at RT and incubated with primary antibodies overnight at 4 °C inside a dark chamber (LC3 antibody, #2775S, Cell Signaling Technology^®^, 1/300, rabbit; PDHA1 antibody, ab110330, Abcam, 1/200, mouse) in 0.05% saponin and Dako Antibody Diluent with Background Reducing Components as solvent. After washing, samples were incubated with secondary antibodies (1 h at RT, anti-rabbit Cy3, 1/300; Alexa Fluor 488 donkey anti-mouse IgG A21202 Invitrogen, 1/300) and acid nucleic marker DRAQ5TM (DR50200, BioStatus, Leicestershire, UK), washed and mounted in 5 µL of Fluoromount-G^®^ (0100-01, Southern Biotech, Birmingham, AL, USA). Pictures, ten random fields per sample, were taken at the confocal microscope Leica TCS SPE with the 60× oil objective. 

### 2.7. Immunoblot Analysis 

Cell lysates were prepared in RIPA buffer plus proteinase inhibitors. Samples containing (20 µg) were separated by 10–15% SDS-PAGE, transferred to nitrocellulose membranes, blocked in 5% nonfat milk for 1h at RT and incubated overnight at 4 °C with the primary antibodies: MFN2 (H-68, Santa Cruz, sc-50331, dilution 1:1000, rabbit); Optineurin (C-2, Santa Cruz, sc-166576, dilution 1:1000, mouse); PINK1 (BC100-494, Novus Biologicals, dilution 1:2000, rabbit); Parkin (PRK8, ab77924, Abcam, dilution 1:2000, mouse); β-Actin (Sigma-Aldrich, A3854, dilution 1:40,000 conjugated to HRP). Secondary antibody incubation was performed for 1 h at RT using anti-mouse (m-IgGκ BP-HRP sc-516102, Santa Cruz, 1:10,000) and anti-rabbit (goat anti-rabbit IgG-HRP sc-2054, Santa Cruz, 1:10,000). Proteins were detected using ECL western blotting substrate (Pierce, Waltham, MA, USA), Clarity and Clarity Max (Bio-Rad, Hercules, CA, USA) and ChemiDoc Imaging System (Bio-Rad).

### 2.8. Statistical Analyses

Results are expressed as mean ± standard deviation and *n* = 3, unless indicated. Statistical comparisons were usually performed using unpaired 2-tailed Student’s *t*-test. A *p*-value less than 0.05 was considered significant.

## 3. Results

### 3.1. Anti-Tumor Activity of Multikinase Inhibitors on Liver Cancer Cells Is Affected by Redox Status

**Figure 1:**
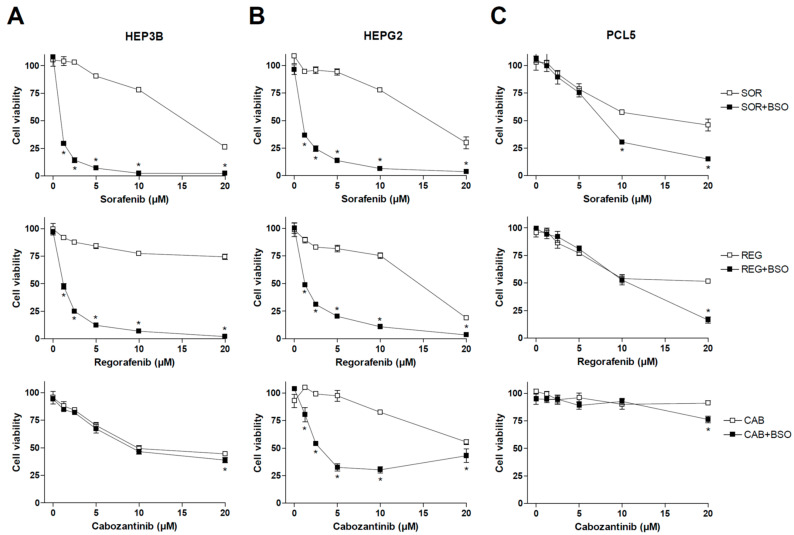
Effect of BSO administration on liver tumor cell lines treated with MKIs. Hep3B (A), HepG2 (B) and PLC5 (C) cells were exposed to increasing doses of sorafenib, regorafenib and cabozantinib for 20 h after incubation with vehicle (PBS) or BSO (1 mM), inhibitor of GSH synthesis, and cell viability quantified by MTT (*n* = 3). * *p* < 0.05 vs. control.

Previous works have demonstrated that the main MKIs used in liver cancer, such as sorafenib and regorafenib, share mitochondrial-dependent cytotoxicity. Therefore, we decided to test if changes in GSH levels, a critical mitochondrial antioxidant against ROS damage in the liver, could affect the anti-tumor activity of MKIs used in HCC therapy such as sorafenib, regorafenib and cabozantinib. To do so, representative hepatoma cell lines were treated with sorafenib, MKI administered in first line for HCC patients, as well as regorafenib and cabozantinib, recommended in second- and third-line therapy (Figure 1), under regular culture conditions or after pre-treatment with BSO, an inhibitor of GSH synthesis that effectively depletes its concentration in vitro and in vivo [29,30]. 

As observed above, sorafenib and regorafenib were very sensitive to GSH depletion, exhibiting cell death clearly potentiated by BSO administration. However, no significant changes were observed in cabozantinib-treated Hep3B cells after diminishing GSH levels. We also tested BSO effect on MKI efficacy in HepG2 cells, observing once again a potent synergy in sorafenib and regorafenib action. In this case, cabozantinib cytotoxicity was also potentiated by GSH reduction. 

Finally, we tested MKIs and BSO in PLC5 cells, another typical hepatoma cell line, finding again sensitization to sorafenib and regorafenib by BSO addition, but at higher doses, not frequently reached in patient treatment, than previously observed in HepG2 and Hep3B cell lines. Once again, cabozantinib toxicity was not clearly affected by GSH modulation in PLC5 cells, suggesting a lower capacity of cabozantinib to generate ROS-dependent death (Figure 1). 

### 3.2. Glutathione Reduction Potentiates Early ROS Production by Multikinase Inhibitors and BH3-Mimetics on Liver Cancer Cells

**Figure 2:**
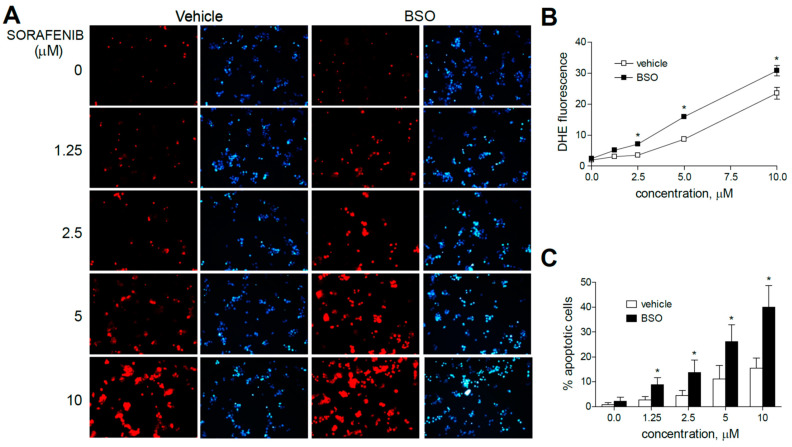
Superoxide production, increased by sorafenib in liver cancer cells, is enhanced by GSH reduction. (**A**) Representative fluorescence images (*n* = 10) after superoxide detection (DHE, red) and nuclear (Hoechst 33258, blue) staining of HepG2 cells at increasing concentrations of sorafenib. (**B**) Quantification of DHE fluorescence in sorafenib-treated cells was analyzed using Image J software. (**C**) Percentage of apoptotic nuclei was measured (*n* = 3). * *p* < 0.05 vs. control.

**Figure 3:**
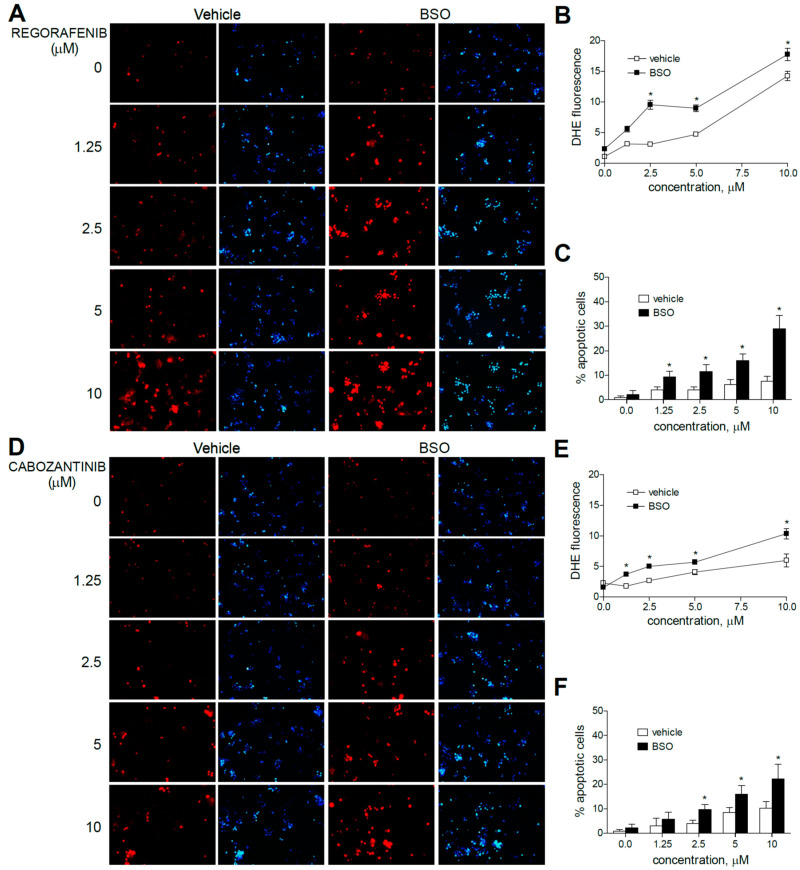
Superoxide production, increased by MKI treatment, is enhanced by GSH reduction. Representative fluorescence images (*n* = 10) after superoxide detection (DHE, red) and nuclear (Hoechst 33258, blue) staining of HepG2 cells after regorafenib (**A**) or cabozantinib (**D**) at increasing concentrations. (**B**,**E**) Quantification of DHE fluorescence in MKI-treated cells was measured using Image J software. (**C**,**F**) Number of apoptotic cells was counted (*n* = 3). * *p* < 0.05 vs. control.

To verify that the increased anti-tumoral effect of these MKIs observed after BSO treatments was preceded by early mitochondrial ROS production, liver cancer cells were analyzed by dihydroethidium (DHE) staining. DHE oxidation, frequently used for cellular and mitochondrial O_2_^•−^ detection, was visualized by fluorescence microscopy in HepG2 cells after three hours of MKI administration (sorafenib, regorafenib or cabozantinib) and/or previous GSH reduction with BSO (Figure 2 and Figure 3).

Sorafenib increased DHE staining at all concentration (1.25 to 10 µM) ranges in HepG2 cells, even at the low micromolar levels similar to those reached during HCC systemic treatment, in agreement with the specific mitochondrial superoxide production upon sorafenib exposure previously shown [31]. 

Moreover, the pre-administration of BSO to deplete GSH levels clearly increased the superoxide production induced by sorafenib in all the hepatoma cell lines tested. Similar effects were observed after BSO challenge and regorafenib-treated cells, although ROS generation was mainly observed in monotherapy at the higher MKI concentrations used. As observed in sorafenib-treated cells, BSO potentiated the apoptosis induced by regorafenib treatment (Figure 3A–C).

Regarding cabozantinib, another tyrosine kinase inhibitor with anti c-MET and AXL activities [6,7], ROS production was less evident at low concentrations. However, BSO pre-treatment was effective in HepG2 cells in increasing superoxide staining, consistent with the potentiation in cabozantinib-induced cell death only observed in this specific cell line (Figure 3D–F).

**Figure 4:**
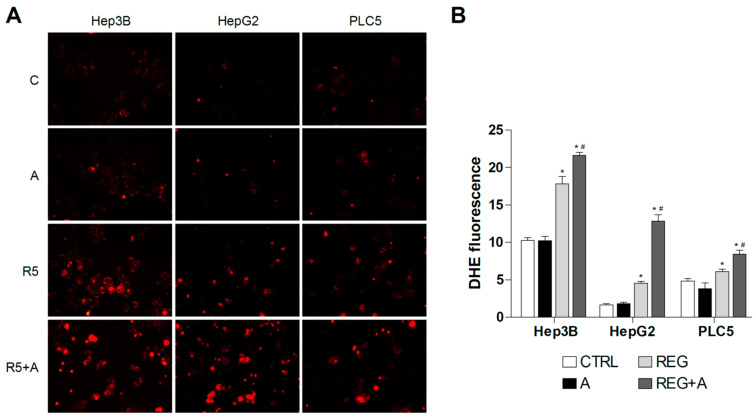
Superoxide production by regorafenib treatment was enhanced in liver cancer cell lines after co-administration with a BH3 mimetic. (**A**) Representative fluorescence images (*n* = 10) of DHE (red) staining in HepG2, Hep3B and PLC5 cells receiving regorafenib treatment (R, 5 µM) in combination with or without the BCL-xL inhibitor A-1331852 (A, 0.1 µM) for 4 h. (**B**) Quantification of DHE fluorescence in cells was measured using Image J software. (*n* = 3). * *p* < 0.05 vs. control, # *p* < 0.05 vs. regorafenib-treated cells.

Due to the mitochondrial effects of sorafenib and regorafenib, the co-administration of BH3-mimetics is now under clinical trial, and similar strategies are under biomedical scrutiny [32,33]. To identify if mitochondrial ROS production may play a role in this action, DHE staining was analyzed in hepatoma cells treated with MKI plus BCL-2 inhibitors. First, we treated liver cancer cell lines with regorafenib and the specific BCL-xL inhibitor A-1331852, a BH3-mimetic that greatly potentiates regorafenib anti-tumor activity, to verify if ROS production is modified during BH3-mimetic sensitization. As observed bellow (Figure 4), regorafenib production of mitochondrial ROS was clearly enhanced after A-1331852 addition in all liver cell lines tested.

### 3.3. Antioxidants May Protect Liver Cancer Cells against Sorafenib/Regorafenib-Based Anti-Cancer Therapies 

**Figure 5:**
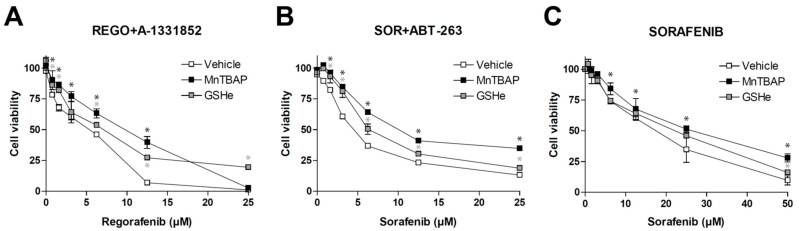
Antioxidants diminish the efficacy of sorafenib/regorafenib-based anti-cancer therapies. MTT assays in Hep3B cells were performed to evaluate potential cell death protection by SOD mimetic MnTBAP and GSHe, a cell permeable GSH supplier, in front of different experimental cancer therapies proposed for HCC treatment. (**A**) increasing doses of regorafenib in combination with the BCL-xL inhibitor A-1331852 (A, 0.1 µM). (**B**) increasing doses of sorafenib in combination with the BCL-2/BCL-xL inhibitor navitoclax (ABT-263) and (**C**) sorafenib alone (*n* = 3). * *p* < 0.05 vs. control cells.

After establishing that different MKIs such as sorafenib or regorafenib, alone or combined with other anti-tumor compounds such as BH3-mimetics, are generating ROS that affect tumor growth, we checked the potential influence of well-known antioxidants. As previously observed [15], regorafenib/A-1331852 action against hepatoma cells was potent and seriously reduced in the presence of MnTBAP or GSHe (Figure 5A), suggesting that antioxidant administration may jeopardize the efficacy of this therapy. 

As expected, the antioxidant protection was not exclusive for this therapy. ROS blockage using MnTBAP or GSHe was also effective in reducing the potent anti-tumoral effect of sorafenib plus navitoclax (Figure 5B), a chemotherapeutic combination used in an on-going clinical trial for treating US patients with relapsed or refractory solid tumors (NCT02143401). 

Finally, since sorafenib was the MKI that showed the greatest capacity to generate ROS (Figure 2), we also tested if these antioxidants were able to reduce cell death caused by sorafenib in monotherapy.

As observed with the combination therapies, MnTBAP and GSHe were also able to reduce sorafenib activity against liver cancer cells (Figure 5C). Although the effect was only detected at high sorafenib doses, it confirms that this feature may be shared by other anti-cancer strategies applied in the clinic.

### 3.4. Mitochondrial ROS Production Controls Tumor Growth in HCC Spheroids 

To validate our in vitro results in an HCC model better resembling human liver cancer than traditional monolayer cultures, we used Hep3B spheroids. Tumor spheroids were treated with sorafenib and/or ROS modulators for several days. 

**Figure 6:**
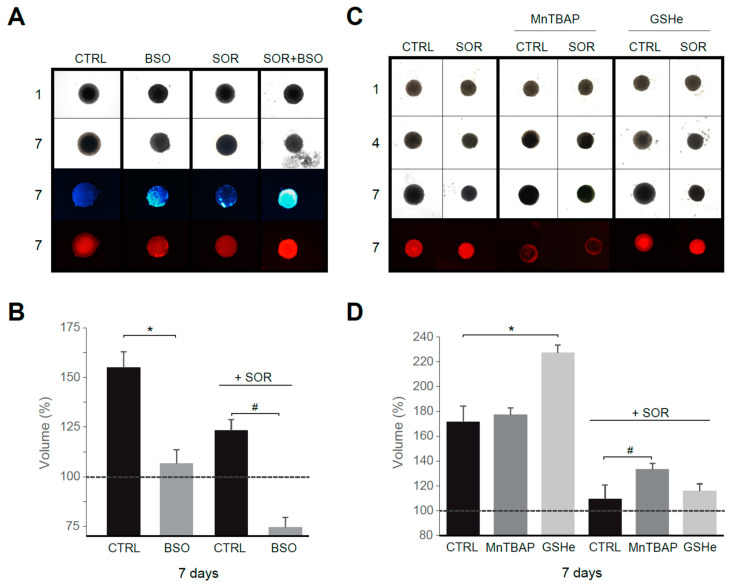
Superoxide reduction in sorafenib treatment favors tumor growth in a 3D spheroid model. (**A**) Hep3B spheroids were seeded (day 1) and treated with vehicle, sorafenib (S, 2.5 μM) and/or BSO (1 mM) for six days. Third row, Hoechst staining. Fourth row, DHE staining. (**B**) Spheroid growth was monitored (*n* = 3). (**C**) Effect of MnTBAP and GSHe on spheroid growth (*n* = 3). (**D**) Volume quantification. * *p* < 0.05 vs. control cells, # *p* < 0.05 vs. sorafenib-treated cells.

First, using sorafenib at a concentration in the range reached in serum during chemotherapy, we observed that treatment with the GSH synthesis inhibitor BSO reduced tumor growth, particularly in sorafenib-treated spheroids (Figure 6A,B). In fact, increased ROS production, detectable after BSO treatment, was potentiated by sorafenib addition, as visualized with DHE staining. In parallel, an increased number of apoptotic cells were detected in Hep3B spheroids under sorafenib plus BSO treatment, as denoted by in vivo Hoechst staining. Interestingly, antioxidant supplementation not only avoided tumor reduction in sorafenib/navitoclax-treated spheroids, principally after superoxide reduction with MnTBAP, but also increased tumor growth (Figure 6C), particularly after GSH supplementation using GSHe as quantified in Figure 6D. 

Therefore, the intracellular antioxidant levels modulate tumor growth under chemotherapy exposure in a 3D model of cancer that could anticipate an in vivo impact for antioxidant supplements.

### 3.5. MKI-Based HCC Therapy Induces Mitochondrial ROS Promoting Mitophagy 

**Figure 7:**
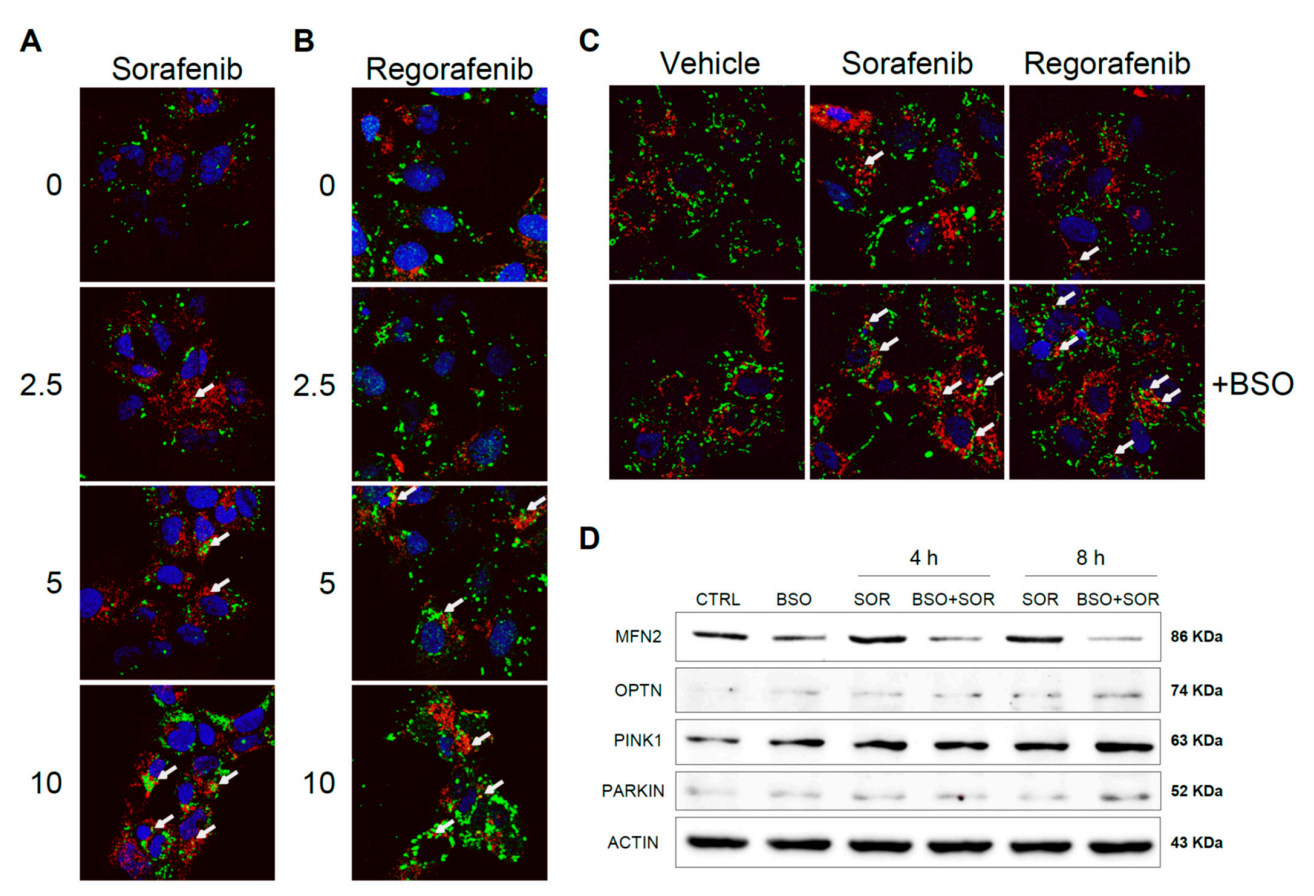
Sorafenib and regorafenib induced mitochondrial-ROS dependent mitophagy in hepatoma cells. Hep3B cells were treated with increasing doses of sorafenib (**A**) or regorafenib (**B**), stained with LC3 (red) and PDHA1 (green) antibodies and visualized by confocal microscopy after 16 h. Representative images of 12 independent random fields. (**C**) Hep3B cells pre-incubated with vehicle or BSO were treated with sorafenib (2.5 μM) or regorafenib (2.5 μM) and visualized as before. (**D**) Hep3B cells, incubated with vehicle or BSO, were treated with sorafenib (2.5 μM) at different times and different mitophagy-related proteins were analyzed by western blot. Representative images (*n* = 3).

Sorafenib interaction with subunits of the mitochondrial respiratory system generates ROS that may promote mitochondrial damage and mitophagy [25,26]. To test if antioxidants and the redox status of hepatoma cells may alter chemotherapy-induced mitophagy, Hep3B cells were exposed to increasing doses of sorafenib and regorafenib, and the effects of increased mROS production were analyzed after BSO exposure. Using a mitochondrial marker such as PDHA1 (green) in combination with the autophagy protein marker LC3 (red), after sorafenib treatment we visualized a dose-dependent increase in LC3 content, consistent with autophagy induction and the appearance of yellow dots indicating co-localization of LC3 and mitochondria (Figure 7A). 

Similar evidence of mitophagy was also observed in regorafenib-treated hepatoma cells (Figure 7B). Of note, the reduction in GSH by BSO pretreatment potentiated mitophagy at low micromolar sorafenib/regorafenib doses, suggesting that oxidative mitochondrial damage by MKI exposure promotes redox-dependent induction of mitophagy (Figure 7C). To verify this point, we analyzed by western blot the levels of relevant mitophagy-related proteins in Hep3B cells treated with sorafenib, evaluating changes after GSH reduction (Figure 7D).

As previously reported, sorafenib increased the cellular amount of PINK1 and Parkin [25,26], promoting the elimination of damaged mitochondria, and the pro-oxidant conditions induced in our experiments by BSO exposure potentiated this effect. Moreover, Parkin-related mitofusin 2 (MFN2) ubiquitination and proteasomal degradation [34] seem to be potentiated by GSH restriction. Stress-induced phosphorylation and proteasomal elimination of MFN2 results in mitochondrial fragmentation, a necessary event for mitophagy induction and enhanced apoptotic cell death [35]. Since MFN2 levels were decreased upon sorafenib exposure in BSO-treated cells, oxidative-induced blockage of mitochondrial fusion could be taking place. Finally, optineurin accumulation and lack of mitochondrial targeting has been described in ROS-induced mitophagy [36]. Since we found increased optineurin in sorafenib-treated hepatoma cells, particularly after BSO pre-incubation, it could be indicative of its MKI-dependent cytosolic accumulation. 

Therefore, these experiments suggest that the mitochondrial damage caused by MKIs promotes mitophagy induction, while a pro-oxidative mitochondrial condition alters mitophagy progression and changes mitochondrial dynamics, in line with recent data [37]. Knowing whether this effect is an important contributor to BSO-induced cell death in chemotherapy would provide novel insights into mitochondria-dependent apoptosis and cancer therapy, a point that would require additional research.

## 4. Discussion

Over-the-counter dietary and nutritional supplements are commonly consumed by the general public, initially as a remedy for medical problems, but increasingly as simple additions to our diet for the alleged prevention of disease. Although nutritional supplements could be beneficial in several settings, their unrestricted intake may also have deleterious effects on human health, which are of special concern to cancer patients [38,39]. Antioxidants play an important role in maintaining cellular integrity against physiological and pathological oxidative stress, which is normally well controlled in healthy individuals [21,22,40,41]. Patients under cancer chemotherapy are conscious that their bodies are under distress and may be prone to taking supplements, particularly since no negative side-effects are expected from them. In fact, the antiangiogenic action of MKIs is well known among physicians and researchers; however, the role of ROS, and specifically of mitochondrial ROS, on MKI efficacy has not been commonly recognized. In this sense, it is important to ponder the relevance of mitochondrial oxidative stress in MKI action against liver cancer and to question the appropriateness of antioxidant supplements during MKI treatments.

Previous research has indicated that ROS are generated from the cellular action of sorafenib or regorafenib, and superoxide from mitochondria was pointed as a probable source. Our work indicates that mitochondrial ROS are common to several MKIs, including cabozantinib, in specific hepatoma cell lines as observed at higher doses and clearly evidenced after reducing antioxidant protection by BSO pre-administration. In fact, depleting GSH levels sensitized against sorafenib, regorafenib and even cabozantinib, in different hepatoma cell lines, supports the key role of ROS in MKI anti-cancer activity. Of note, the increase in mitochondrial ROS after MKI therapy is also common to other successful strategies [42,43,44], as we observed after regorafenib co-treatment with the BH3 mimetic A-1331852. Similarly, it may suggest that other compounds able to generate mitochondrial stress in cancer cells might be worthy of combination with MKI therapy in HCC treatment [42]. 

Once it was demonstrated that ROS induction by MKIs participates in the killing of cancer cells, we wanted to test if derivatives of GSH and SOD, two of the compounds more frequently recommended as antioxidant dietary supplements, could modify MKI action in hepatoma cell lines. To do so, we used the SOD mimetic MnTBAP or GSH ester (GSHe), since they both have been intensively used in vitro and in vivo [24,29,30,40,41], and they can easily target intracellular ROS even in mitochondrial compartments. Noteworthy, MnTBAP and GSHe diminished the efficacy of sorafenib and regorafenib, not only alone but also combined with BH3-mimetics, emphasizing the relevant participation of ROS in cancer therapy. Similar behavior was detected in 3D tumor spheroids, highlighting the potential problems associated with antioxidant intake during MKI therapy. It is worth remarking that this perturbing effect was fortunately not shared by all antioxidants tested. For instance, no significant protection from death was observed after administering Trolox, a vitamin E analog, or MitoQ (data not shown), a mitochondrial-targeted antioxidant that frequently protects against mitochondrial damage [45,46]. Regarding MitoQ, this TPP+-conjugated antioxidant selectively concentrates in the mitochondria and prevents mitochondrial oxidative damage, being frequently bought for sport practitioners and the public in general. However, MitoQ did not potentiate the toxicity of MKIs in liver cancer cell lines. As a possible explanation, TPP+-conjugated antioxidants penetrate the mitochondria leaded by the mitochondrial membrane potential (MMP) [47], and sorafenib or regorafenib quickly and strongly decrease MMP in hepatoma cells, which could prevent TPP+ mitochondrial entry. 

Finally, we wanted to verify mitophagy participation in MKI action since mitochondrial damage by ROS producing drugs is becoming an interesting subject modulated by the redox state, with potential antioxidant participation. Our data support that the mitochondrial damaging effect of MKIs, such as sorafenib and regorafenib, is promoting mitophagy in hepatoma cells, being the PINK1/Parkin signaling pathway clearly enhanced by modifying the antioxidant defense, as BSO pre-incubation does. Although clearance of damaged mitochondria by mitophagy is thought to mediate drug resistance in cancer cells, excessive mitochondrial clearance may induce cell metabolic disorders and cell death [48], in line with our previous results in sorafenib resistant cells [11]. Interestingly, mitochondrial fission-stimulated ROS production on chemotherapy is proposed as a reasonable target for pharmacological stimulation of mitochondrial dynamics that can benefit cancer patients with solid tumors [49]. Therefore, it is tempting to speculate that not only MKIs but also other autophagy/mitophagy-based therapies for cancer could be affected by oxidative stress and antioxidant supplementation. 

## 5. Conclusions

Patients and physicians must be conscious that MKI-based therapies are producing mitochondrial ROS with an important role in the anti-cancer efficacy of the drugs. In particular, dietary supplements with potent antioxidant properties may not be recommended for individuals taking sorafenib or regorafenib for liver cancer treatment. This precaution should be extended for other chemotherapeutic compounds, since the absence of strong evidence indicating ROS involvement in the anti-tumor action, as it happens with cabozantinib reported studies, does not necessarily guarantee the lack of side-effects on specific cancer cells. On the other side, therapies combining pro-oxidant compounds with MKIs should be pursued since cellular redox status modulates MKI effectiveness and may affect therapies with associated autophagy/mitophagy induction.

## Data Availability

Data is contained within the article.
